# Immunogenic chemotherapy sensitizes RAS-mutated colorectal cancers to immune checkpoint inhibitors

**DOI:** 10.1080/2162402X.2023.2272352

**Published:** 2023-11-09

**Authors:** Lucillia Bezu, Oliver Kepp, Guido Kroemer

**Affiliations:** a Equipe Labellisée Par La Ligue Contre Le Cancer, Université Paris Cité, Sorbonne Université, INSERM UMR1138, Centre de Recherche des Cordeliers, Institut Universitaire de France, Paris, France; b Metabolomics and Cell Biology Platforms, Villejuif, France; c Département d’Anesthésie, Chirurgie et Interventionnel, Gustave Roussy, Villejuif, France; d Department of Biology, Institut du Cancer Paris CARPEM, Hôpital Européen Georges Pompidou, AP-HP, Paris, France

**Keywords:** Cancer, immunogenic cell death, immunotherapy

## Abstract

Recent clinical trials have compared the use of different chemotherapeutic regimens as “immune induction therapies” to sensitize cancers to immune checkpoint inhibitors (ICI). Cytotoxic drugs reputed to be inducers of immunogenic cell death (ICD) appeared to be particularly efficient for this purpose. A trial published in Nature Medicine by Thibaudin et al. reveals the capacity of oxaliplatin-based chemotherapy to sensitize RAS-mutant unresectable metastatic colorectal cancer to ICIs blocking CTLA-4 and PD-L1.

## Main text

Immunogenic cell death (ICD) is a specific modality of cytolysis triggered by agents with immunoactivatory properties. In brief, ICD-inducing therapies, trigger a premortem stress in tumor cells that promotes the emission of a specific array of danger-associated molecular patterns (DAMPs). Partial endoplasmic reticulum (ER) stress, characterized by the phosphorylation of eukaryotic initiation factor 2 subunit 1 (eIF2α), induces the translocation of endoplasmic reticulum (ER) chaperones including calreticulin (CALR) to the plasma membrane where they act as ligands for CD91 expressed on dendritic cells (DCs) thus serving as an “eat-me” signal to stimulate DC-mediated phagocytosis. Furthermore, the onset of autophagy in cancer cells undergoing ICD facilitates the lysosomal liberation of ATP that in turn can ligate purinergic receptor P2X 7(P2RX7), thus serving as a chemoattractant guiding DCs to the tumor bed. The final homing of DCs is further supported by the release of annexin A1 by cancer cells, which interacts with formyl peptide receptor 1 (FPR1) located on the surface of DCs, thus facilitating their interaction with tumor debris. During the course of ICD tumor cells also secrete type I interferon (IFN), which exerts autocrine effects promoting the synthesis of CXCL10 as well as paracrine effects reinforcing the chemotaxis of DCs. Moreover, tumor cells succumbing to ICD release high-mobility group box 1 (HMGB1), which acts on toll-like receptor 4 (TLR4) and triggers DCs maturation. Mature DCs are endowed with the capability to process and expose tumor antigens to T lymphocytes. Ultimately, activated cytotoxic T lymphocytes (CTLs) induce an IFN-γ-mediated killing of residual malignant cells and establish immune memory preventing cancer recurrence.^1^

Different pharmacological classes of compounds have been revealed to be particularly efficient inducers of ICD, as this applies to some conventional chemotherapeutic agents including anthracyclines, oxaliplatin, taxanes and vinca alkaloids, as well as to targeted agents such as the tyrosine kinase inhibitor crizotinib, the proteasome inhibitor bortezomib and cardiotonic glycosides such as digoxin, digitoxin and oleandrin. On the contrary, other clinically-approved anticancer agents such as the platinum salt cisplatin are poor ICD inducers.^1^

Often, chemotherapy is combined with immunotherapy (usually targeting the PD-1/PD-L1 interaction) to obtain chemo-immunotherapeutic effects in cancer patients.^2,3^ Apparently, conventional chemotherapeutics that are endowed with the capacity to induce ICD can render the tumor microenvironment sensitive to immune checkpoint inhibition (ICI) by upregulating PD-L1 expression and enhancing CD8^+^ T cell infiltration,^4,5^ thus *de facto* converting a cold tumor microenvironment into a hot one. These preclinical findings were recently confirmed in the Checkmate 649 randomized, multicenter, open-label trial.^6^ In this study involving 1581 patients with unresectable gastric carcinomas, overall (OS) and progression-free survival (PFS) were significantly improved after treatment with the combination of oxaliplatin plus nivolumab (anti-PD-1) compared to oxaliplatin alone (HR = 0.71 [98.4%, CI 0.59–0.86]; *p* <.0001 and HR = 0.68 [98%, CI 0.56–0.81]; *p* <.0001, respectively). In contrast, the Keynote-062 study, a comparable randomized phase III trial also targeting gastric cancers failed to observe a benefit when the non-ICD inducer cisplatin was combined with pembrolizumab (also an anti-PD-1 antibody), highlighting the fact that only ICD inducers can promote synergistic effects with immunotherapy. Nevertheless, it should be noted that the chemoimmunotherapy group presented more grade 3 to 5 treatment-related adverse events (AEs) than the control group receiving chemotherapy alone.^7^

Results from the randomized phase IIb controlled ALICE trial including 68 patients with metastatic triple negative breast cancer (mTNBC) demonstrated that the efficacy of immunogenic chemotherapy (doxorubicin plus cyclophosphamide) could be enhanced by combination with atezolizumab (anti-PD-L1). Thus, the chemoimmunotherapy combination significantly improved PFS as compared to chemotherapy alone (median 4.3 months *vs* 3.5 months; HR = 0.57 [95% CI, 0.33–0.99]; log-rank *p* =.047).^8^ Intriguingly, in the phase II TONIC trial enrolling 67 patients with mTNBCs for the evaluation of short-term induction treatments with low-dose irradiation, cyclophosphamide, cisplatin or doxorubicin prior to nivolumab, the best objective response rate (ORR) was observed with the ICD-inducer doxorubicin (ORR = 35% [95% CI, 14.2–61.7]). Indeed, in the doxorubicin/nivolumab arm, immune-related genes linked to PD-1/PD-L1 blockade were upregulated, and cytolytic T cells signaling pathways were triggered within the tumor bed. Consistently a significantly higher number of intratumoral T cells was found in the doxorubicin/nivolumab group as compared to controls.^9^

Finally, in the recently published phase Ib/II MEDITREME trial of Ghiringhelli et al., 57 patients with unresectable RAS-mutated metastatic colorectal cancers received durvalumab (anti-PD-L1) plus tremelimumab (anti-CTLA-4) together with the ICD inducer oxaliplatin and 5-fluorouracil for 3 months, followed by durvalumab alone for 9 months. This combination induced grade 1/2 and 3/4 AEs in 98% of patients leading to a treatment discontinuation in 7 patients. Despite these limitations, significant benefits in terms of survival were observed. The PFS at 3 months was 90.7% [95% CI, 79.2–96], the response rate was 64.5% and OS at 6-month was 95.8% [95% CI, 84.3–98.9]. An increase in CTLA-4 expression in the tumor was associated with significantly better responses (HR = 0.22 [95% CI, 0.09–0.53], *p* =.001). In sum, the association of immunogenic chemotherapy with double checkpoint blockade substantially potentiated the immune control of RAS-mutated colorectal tumors, thus offering a putative curative treatment for unresectable cancers of this type.^10^ Table 1.

**Table 1.** Clinical trials investigating efficacy and safety of chemo-immunotherapy.

Trial	Indication	Chemotherapy	Immunotherapy	Outcome	Ref.
Checkmate 649	Non-HER2-positive gastric, gastro-esophageal, or esophageal adenocarcinoma	Oxaliplatin + capecitabin or 5-fluorouracil + leucovorin	Nivolumab	Benefits in OS and PFS compared to chemotherapy alone	^6^
Keynote 062	Untreated, advanced gastric or gastro-esophageal cancer	Cisplatin + capecitabin or 5-fluorouracil	Pembrolizumab	No benefits in OS in patients with CPS of 1 or greater or CPS of 10 or greater or in PFS in patients with CPS of 1 or greater	^7^
ALICE	Metastatic TNBC	Doxorubicin + cyclophosphamide	Atezolizumab	Improved PFS; proportion without progression or death at 15-month was 14.7% for chemoimmunotherapy *vs* 0% for control group	^8^
TONIC	Metastatic TNBC	Irradiation or cyclophosphamide or cisplatin or doxorubicin	Nivolumab	No induction (ORR = 17%); Irradiation (ORR = 8%); cyclophosphamide (ORR = 8%); cisplatin (ORR = 23%); doxorubicin (ORR = 35%)	^9^
MEDITREME	Metastatic RAS-mutated CRC	Oxaliplatin + 5-fluorouracil	Durvalumab + Tremelimumab	PFS at 3-, 6-, 12- and 24-month was respectively 90.7%; 60.4%; 26.9%; 6.7% OS at 6-, 12- and 24-month was respectively 95.8%; 81.1%; 57.6%.	^10^

*Abbreviations: CI, confidence interval; CPS, combined positive score; CRC, colorectal cancer; HR, hazard ratio; ICI, immune checkpoint inhibitor; ORR, objective response rate; OS, overall survival; PFS, progression-free survival; TNBC, triple negative breast cancer*.

In conclusion, accumulating clinical data suggest that the induction of ICD sensitizes cancer to the effects of subsequent immunotherapy with ICIs. Consensus guidelines are urgently required to design specific ICD-immunotherapy regimens that improve oncological outcome but limit severe adverse events.

## Abbreviations

AE: adverse event
CTLA-4: cytotoxic T-lymphocytes-associated protein-4
DAMPs: danger-associated molecular patterns
DC: dendritic cell
eIF2alpha: eukaryotic Initiation Factor 2 alpha
ER: endoplasmic reticulum
HMGB1: High-Mobility Group Box 1
ICD: immunogenic cell death
ICI: immune checkpoint inhibitors
IFN: interferon
OS: overall survival
PD-1: programmed cell death protein 1
PD-L1: programmed cell death 1 ligand 1
PFS: progression-free survival
TNBC: triple negative breast cancer
